# DNA_3′_pp_5′_G de-capping activity of aprataxin: effect of cap nucleoside analogs and structural basis for guanosine recognition

**DOI:** 10.1093/nar/gkv501

**Published:** 2015-05-24

**Authors:** Mathieu Chauleau, Agata Jacewicz, Stewart Shuman

**Affiliations:** Molecular Biology Program, Sloan-Kettering Institute, New York, NY 10065, USA

## Abstract

DNA_3′_pp_5′_G caps synthesized by the 3′-PO_4_/5′-OH ligase RtcB have a strong impact on enzymatic reactions at DNA 3′-OH ends. Aprataxin, an enzyme that repairs A_5′_pp_5′_DNA ends formed during abortive ligation by classic 3′-OH/5′-PO_4_ ligases, is also a DNA 3′ de-capping enzyme, converting DNAppG to DNA_3′_p and GMP. By taking advantage of RtcB's ability to utilize certain GTP analogs to synthesize DNAppN caps, we show that aprataxin hydrolyzes inosine and 6-O-methylguanosine caps, but is not adept at removing a deoxyguanosine cap. We report a 1.5 Å crystal structure of aprataxin in a complex with GMP, which reveals that: (i) GMP binds at the same position and in the same *anti* nucleoside conformation as AMP; and (ii) aprataxin makes more extensive nucleobase contacts with guanine than with adenine, via a hydrogen bonding network to the guanine O6, N1, N2 base edge. Alanine mutations of catalytic residues His147 and His149 abolish DNAppG de-capping activity, suggesting that the 3′ de-guanylylation and 5′ de-adenylylation reactions follow the same pathway of nucleotidyl transfer through a covalent aprataxin-(His147)–NMP intermediate. Alanine mutation of Asp63, which coordinates the guanosine ribose hydroxyls, impairs DNAppG de-capping.

## INTRODUCTION

**Figure 1.**
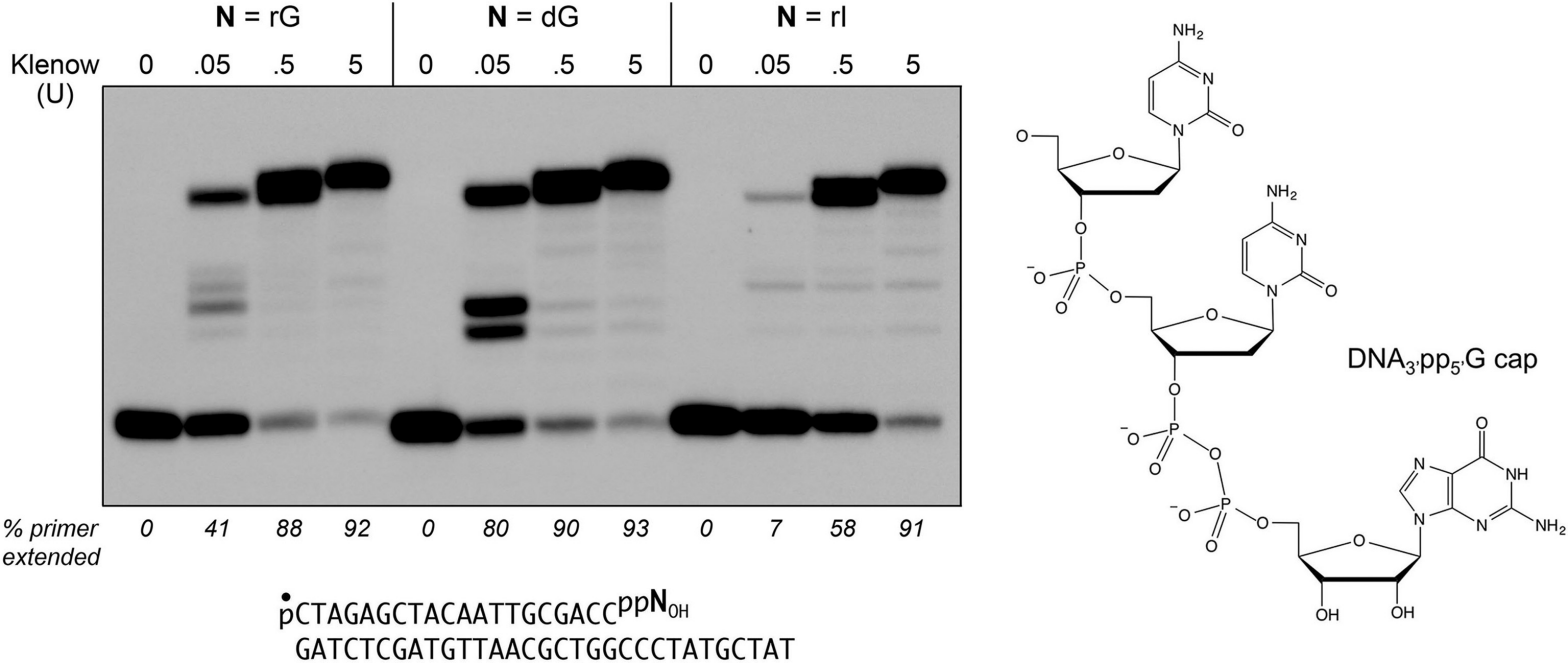
DNA 3′ caps with guanosine nucleotide analogs can prime DNA synthesis. The chemical structure of the DNAppG cap 3′ terminus is shown at *right*. Polymerase reaction mixtures (10 µl) containing 10 mM Tris–HCl (pH 7.9), 50 mM NaCl, 10 mM MgCl_2_, 1 mM DTT, 1 mM each of dATP, dGTP, dCTP and dTTP, 0.2 pmol 5′ ^32^P-labeled pDNAppN_OH_ primer-template (depicted at *bottom* with the ^32^P-label denoted by •) in which N is guanosine, deoxyguanosine, or inosine, and 0, 0.05, 0.5, or 5 U of Klenow Pol I were incubated at 37°C for 20 min. The products were analyzed by urea-PAGE and visualized by autoradiography. The percentages of the input primer strand extended are indicated below the lanes.

The synthesis and repair of DNA rely on enzymatic reactions at 3′-OH ends, which prime nucleotide addition by DNA polymerases and enable 3′-OH/5′-PO_4_ nick sealing by DNA ligases. Repair of ‘dirty’ DNA breaks with 3′-PO_4_ ends classically requires removal of the 3′-PO_4_ (referred to as ‘end-healing’) and/or resection of 3′ nucleotides. The non-canonical RNA ligase *Escherichia coli* RtcB provides an alternative pathway of DNA 3′-PO_4_ end-processing in which RtcB reacts with GTP to form a covalent RtcB-(histidinyl)-GMP intermediate (1,2) and then transfers GMP to a DNA 3′-PO_4_ to form a DNA_3′_pp_5′_G ‘cap’ (3) (Figure 1). Capping protects DNA 3′ ends from resection by free-standing repair exonucleases, from end-healing by polynucleotide 3′ phosphatase, from proofreading by 3′ exonucleases associated with DNA polymerases, and from 3′-OH/5′-PO_4_ nick sealing by DNA ligases (4,5). Yet, the cap is an effective primer for templated DNA synthesis by exemplary members of five DNA polymerase families: A, B, C, X, and Y (4,5). The outcome of cap-primed DNA synthesis is the embedding of a ribonucleotide and a pyrophosphate linkage in the repaired strand. The embedded ribonucleotide 5′ pyrophosphate is refractory to ribonucleotide excision repair initiated by RNase H2 (5).

A key question is whether and how DNA caps might be removed, in the service of either preventing their extension by polymerases or relieving a transient impediment to end-healing. We reported previously that the DNA repair enzyme aprataxin has a DNA 3′ de-capping activity, converting DNAppG to DNA_3′_p and GMP (4). Aprataxin, a member of the histidine triad family of nucleotidyltransferases, has been the focus of much attention because inactivating mutations in the enzyme are the cause of the human neurological disorder ataxia oculomotor apraxia-1 (6,7). Aprataxin is known to de-adenylate abortive A_5′_pp_5′_DNA intermediates that can accumulate when classic DNA ligases attempt to seal DNA 5′-PO_4_ ends at sites of damage or RNA 5′-PO_4_ ends embedded in DNA (8,9). Aprataxin catalyzes the conversion of A_5′_pp_5′_DNA to AMP and pDNA via a covalent enzyme-(histidinyl)–AMP intermediate (9–11). The demonstration of a DNA 3′ de-capping activity inherent in aprataxin (4) raises the prospect that some of the profound neurological consequences of aprataxin deficiency might arise because it is not available to de-guanylylate 3′ capped ends deposited at sites of oxidative DNA damage that generate 3′-PO_4_ breaks.

The dual capacity of aprataxin to de-adenylate a 5′ capped AppDNA and de-guanylate a 3′ capped DNAppG indicate that this enzyme is eclectic in its repair substrate specificity. New questions arise thereby: (i) what is the repertoire of cap nucleobases that aprataxin can remove? (ii) does the DNAppG de-guanylylation reaction occur at the same nucleotide binding site and via the same catalytic mechanism invoked for AppDNA de-adenylylation?

## MATERIALS AND METHODS

### Aprataxin

The *Schizosaccharomyces pombe hnt3* open reading frame encoding aprataxin (SPCC18.09c; Genbank CAA21423) was amplified by polymerase chain reaction (PCR) from a cDNA library and inserted into pET16b. Missense mutations were introduced into the aprataxin gene by PCR amplification with mutagenic primers. The plasmid inserts were sequenced to verify that no unwanted coding changes were acquired during amplification and cloning. Wild-type and mutant His_10_-tagged aprataxins were produced in *E. coli* BL21(DE3) by overnight isopropyl-β-D-thiogalactopyranoside (IPTG) induction of 4-l cultures at 17°C and purified from soluble extracts by Ni-agarose chromatography. Protein concentrations were determined by the BioRad dye binding method with bovine serum albumin as the standard.

### Substrates

The 5′ ^32^P-labeled pDNAp strands were prepared by enzymatic phosphorylation of 20-mer _HO_CTAGAGCTACAATTGCGACCp oligonucleotide by using [γ^32^P]ATP and the phosphatase-dead mutant T4 Pnkp-D167N. The radiolabeled pDNAp strand was gel-purified. The 5′ ^32^P-labeled pDNAppG, pDNAppI, pDNApp(dG) and pDNApp^OMe^G strands were prepared by incubating pDNAp with a 20-fold molar excess of RtcB in the presence of 2 mM MnCl_2_ and 1 mM GTP, ITP, dGTP or 6-OMeGTP for 20 min at 37°C, followed by gel-purification. Primer-templates for DNA polymerase assays were prepared by annealing the radiolabeled pDNAppG, pDNAppI, and pDNApp(dG) primers to a four-fold molar excess of an unlabeled template DNA strand.

### Aprataxin-(33-232)

The open reading frame encoding the catalytic domain (aa 33-232) of fission yeast aprataxin (10,11) was inserted between the BamHI and XhoI sites of vector pET28-His_10_Smt3 so as to fuse the truncated aprataxin polypeptide to an N-terminal His_10_Smt3 tag. The plasmid was transfected into *E. coli* BL21(DE3). A 16-l culture amplified from a single transformant was grown at 37°C in TB broth containing 50 μg/ml kanamycin and 0.4% (v/v) glycerol until the *A*_600_ reached 0.5. The culture was chilled on ice for 30 min, then adjusted to 2% (v/v) ethanol and 0.5 mM IPTG and incubated overnight at 17°C with constant shaking. Cells were harvested by centrifugation and resuspended in buffer A (50 mM Tris–HCl, pH 8.0, 0.25 M NaCl, 10% sucrose) containing two protease inhibitor cocktail tablets (Roche). All subsequent procedures were performed at 4°C. Cells were lysed by sonication and the insoluble material was removed by centrifugation in a Sorvall SLA1500 rotor at 14000 rpm for 45 min. The supernatant was decanted and mixed for 1 h with 5 ml of His60-Ni Superflow resin (Clontech) that had been equilibrated with buffer A. The resin was recovered by centrifugation, resuspended in 50 ml of buffer A, recovered again by centrifugation and resuspended in 50 ml of buffer A. The resin was poured into a column and the adsorbed material was serially step-eluted with buffer B (50 mM Tris–HCl, pH 8.0, 0.25 M NaCl, 10% glycerol) containing 50, 100, 200 and 500 mM imidazole. The elution profile was monitored by sodium dodecyl sulphate-polyacrylamide gel electrophoresis (SDS-PAGE) of the column fractions. His_10_Smt3-Aprataxin-(33-232) was recovered in the 200 and 500 mM imidazole eluates. The peak fractions were pooled, supplemented with Smt3-specific protease Ulp1 (at an aprataxin:Ulp1 ratio of ∼1000:1) and then dialyzed overnight against 2 l of buffer containing 50 mM Tris–HCl, pH 8.0, 100 mM NaCl, 20 mM imidazole. The dialysate was applied to a 5-ml His60-Ni Superflow column that had been equilibrated with buffer B. The tag-free Aprataxin-(33-232) protein was recovered in the flow-through fraction. The protein was concentrated to 10 mg/ml by centrifugal ultrafiltration.

### Crystallization and structure determination

A solution of 0.6 mM Aprataxin-(33-232) and 5 mM GMP was pre-incubated for 15 min at 22°C. Aliquots (2 μl) were then mixed with 2 μl of 100 mM Tris–HCl, pH 8.0, 16% (w/v) PEG-3350, 0.15 M Li_2_SO_4_ and crystals were grown at 22°C by sitting drop vapor diffusion against a reservoir of the precipitant solution. Crystals were cryo-protected by transfer to 100 mM Tris–HCl, pH 8.0, 25% glycerol, 25% (w/v) PEG-3350, 0.15 M Li_2_SO_4_ prior to flash-freezing in liquid nitrogen. Diffraction data at 1.5 Å resolution were collected from a single crystal at the Argonne National Laboratory beamline ID-24-C equipped with Pilatus 6M-F detector. The crystal belonged to space group P2_1_ with unit cell dimensions consistent with two protomers per asymmetric unit, assuming a solvent content of 41%. Indexing, merging and scaling of the diffraction data were performed in XDS (12) and Scala (13). The structure was solved by molecular replacement in Phenix.Phaser (14) using as a search model the previously reported *S. pombe* Aprataxin-(33-332) polypeptide structure (pdb ID 3SZQ), from which DNA, AMP, ions, and water were removed. The structure, comprising two aprataxin protomers, was iteratively refined in Phenix (with NCS restraints) and adjusted manually in Coot (15). The A protomer comprised a continuous polypeptide from amino acids 33 to 231. The B protomer comprised two segments, from 33 to 186 and 193 to 231, punctuated by 6 amino acid gap. Difference density allowed placement of guanosine in the active site of the A protomer and GMP in the active site of the B protomer. The final 1.5 Å model refined to R/R_free_ of 15.7/19.1 with no Ramachandran outliers (Table 1). The coordinates have been deposited in the Protein Data Bank under accession code 4XBA.

**Table 1.** Diffraction data and refinement statistics: aprataxin·GMP

**Diffraction Data**	
Space group	*P2_1_*
Cell dimensions	
*a, b, c* (Å)	52.8, 72.5, 53.9
α, β, γ (°)	90.0, 111.3, 90.0
Wavelength (Å)	0.9795
Resolution (Å)	72.51−1.50 (1.58−1.50)
Reflections	
observed	396152 (57362)
unique	59945 (8750)
R_merge_^a^	0.070 (0.831)
R_meas_^b^	0.076 (0.904)
R_pim_^c^	0.029 (0.353)
I/σI	13.4 (2.5)
CC(1/2) ^d^	0.999 (0.811)
Completeness (%)	99.0 (99.4)
Multiplicity	6.6 (6.6)
Wilson *B*-factor	18.2
	
**Refinement statistics**	
Resolution (Å)	50.26−1.50 (1.52−1.50)
Completeness	98.9 (99.0)
R_work_/R_free_^e^	15.7/19.1
CC* ^d^	1.000 (0.923)
*B*-factors	
protein	22.1
ligands	42.0
solvent	35.1
RMS deviations	
Bond lengths (Å)	0.014
Bond angles (°)	1.569
Ramachandran plot	
Favored (%)	98.0
Outliers	none
	
**Model contents**	
Protomers/ASU	2
Protein residues	392
Water	357
Ligands/Ions	1 GMP, 1 guanosine, 2 Zn^2+^, 1 glycerol
	
**PDB ID**	4XBA

Figures in parentheses refer to data in the highest resolution shell. Data collection statistics are from Scala (CCP4 suite), refinement and geometric statistics come from Phenix.Refine.

(a) R_merge_ describes the spread of multiple observations of the intensity of the unique reflections.

(b) R_meas_ (the redundancy-corrected R_merge_) indicates the precision of an individual intensity measurement independently of the multiplicity of that measurement.

(c) R_pim_ reports the precision of an averaged intensity measurement.

(d) CC(1/2) and CC* are statistics calculated for defining the high-resolution cutoff limits [CC(1/2)] and quality of the diffraction data in the context of the refined structure [CC*].

(e) R*_free_* set consists of ∼5% of data chosen randomly against which structure was not refined.

## RESULTS AND DISCUSSION

### DNA 3′ capping with guanosine nucleotide analogs

*Escherichia coli* RtcB has limited tolerance for GTP analogs as substrates for 3′-PO_4_/5′-OH RNA ligation, whereby it can accept dGTP, ITP, or 6-OMeGTP in lieu of GTP, but cannot utilize adenosine triphosphate (ATP), 6-chloropurine-TP, or 2-aminopurine-TP (11,16). We exploited this tolerance to prepare 5′ ^32^P-labeled 3′ capped DNAppN substrates with either guanosine, deoxyguanosine, or inosine as the cap nucleoside. The pDNAppN strands were annealed to a complementary 31-mer DNA strand to form a primer-template in which the cap nucleobase pairs with a cytosine in the template strand (Figure 1). We compared the ability of *E. coli* DNA Pol I Klenow fragment to extend these primer-templates. Based on the percentages of primer extended at limiting polymerase concentration (0.05 U), we observed that Klenow polymerase was ∼two-fold more effective in utilizing the pp(dG)_OH_ primer than the ppG_OH_ primer (Figure 1). The ppI_OH_ primer was six-fold less effective than ppG_OH_, presumably reflective of the weaker cap I:C base pair *vis à vis* a cap G:C pair. Nonetheless, all three DNAppN_OH_ primers were extended to the end of the template strand at saturating levels of Klenow polymerase (0.5 or 5 U).

### Effect of guanosine analogs on de-capping

**Figure 2.**
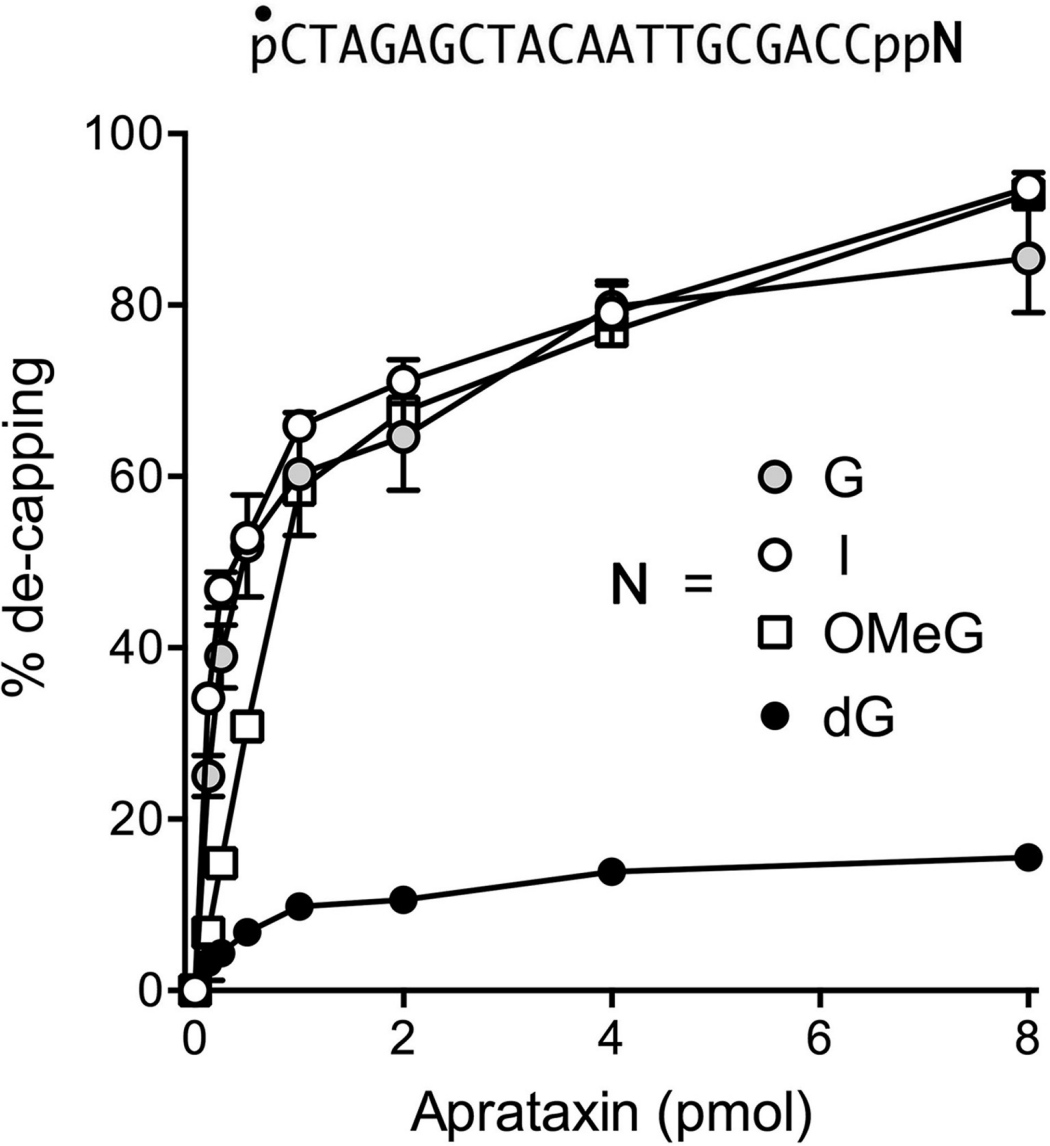
Effect of guanosine analogs on de-capping by aprataxin. Reaction mixtures (10 μl) containing 50 mM Tris–HCl (pH 8.0), 40 mM NaCl, 5 mM EDTA, 0.2 pmol 5′ ^32^P-labeled pDNAppN strand as shown in which N is guanosine, deoxyguanosine, inosine or 6-O-methylguanosine, and 0, 0.125, 0.25, 0.5, 1, 2, 4 or 8 pmol *Schizosaccharomyces pombe* aprataxin were incubated at 30°C for 10 min. The products were analyzed by urea-PAGE. The extents of conversion of pDNAppN to pDNAp are plotted as a function of input aprataxin. Each datum is the average of three independent enzyme titration experiments ±SEM.

We tested the 5′ ^32^P-labeled pDNAppG, pDNAppI, and pDNApp(dG) strands, as well as a pDNApp^OMe^G strand, as substrates for de-capping by recombinant *S*.*pombe* aprataxin. The extents of de-capping as a function of input aprataxin are shown in Figure 2. Specific activity was similar for the pDNAppG and pDNAppI substrates. Whereas activity on the pDNApp^OMe^G substrate was about one-third that of pDNAppG in the linear range of enzyme-dependence, the extent of pDNApp^OMe^G de-capping at saturating aprataxin was similar to that of the pDNAppG and pDNAppI strands. By contrast, pDNApp(dG) was a feeble substrate for de-capping by aprataxin (Figure 2), implying that the cap ribose 2′-OH is a key determinant of aprataxin's de-capping activity.

### Structural basis for cap guanosine recognition by aprataxin

**Figure 3.**
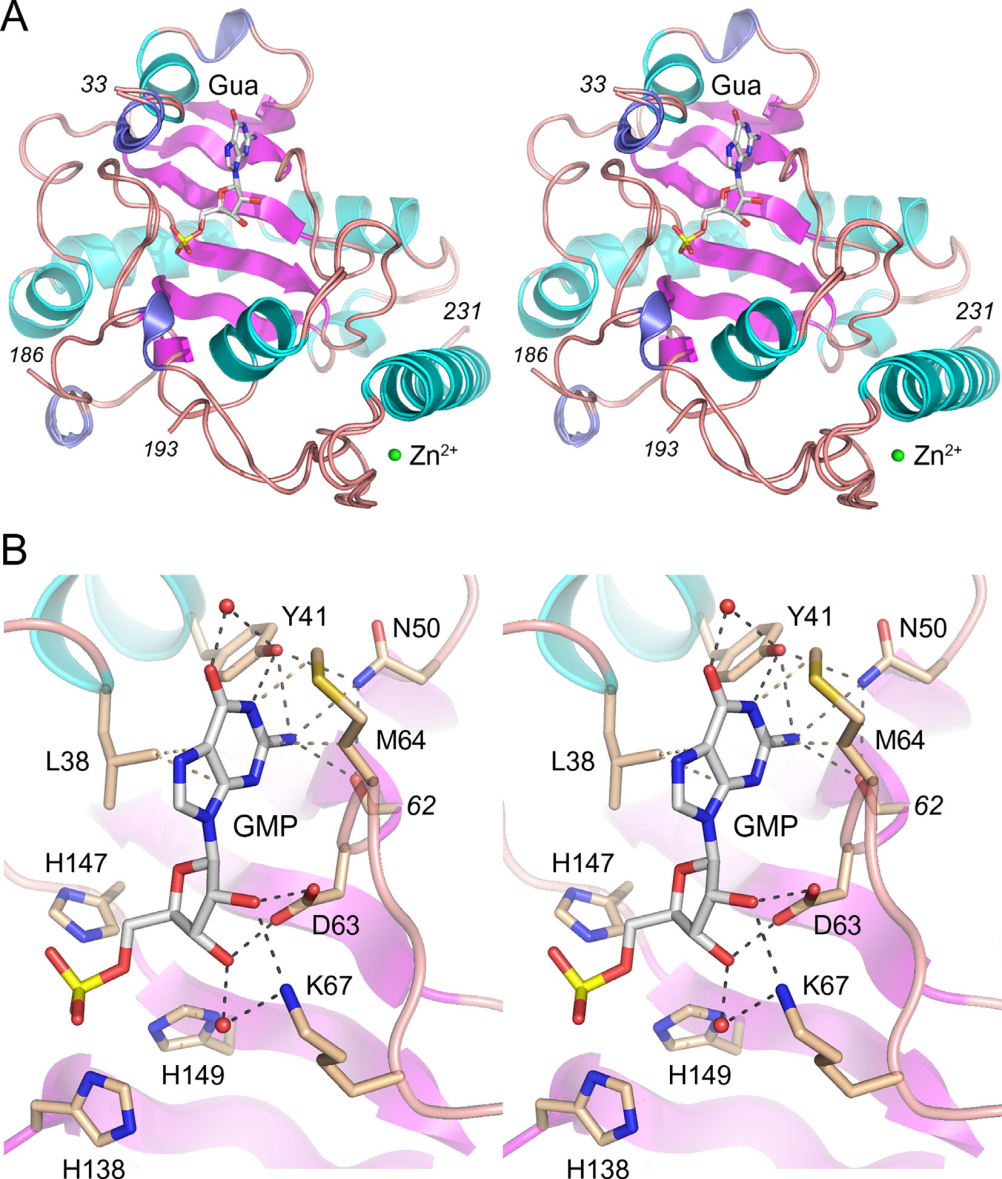
Structural basis for cap guanosine recognition by aprataxin. (**A**) Stereo view of the tertiary structure of the superimposed A and B aprataxin protomers, with β strands, α helices and 3_10_ helices colored magenta, cyan and blue, respectively. Guanosine/GMP in the A/B active sites are shown as stick models. Zn^2+^ is depicted as a green sphere. (**B**) Detailed stereo view of GMP in the active site of the B protomer. Selected amino acids are shown as stick models with beige carbons.

Tumbale *et al*. (9,10) and Gong *et al*. (11) have determined the crystal structures of the catalytic domains of *S. pombe* and human aprataxin at discrete steps along the AppDNA de-adenylylation reaction pathway, e.g. with the hydrolyzed AMP product in the active site or as a vanadate-bridged mimetic of the aprataxin-(histidinyl-Nϵ)-AppDNA transition state. In order to gain insights to how aprataxin recognizes the DNA 3′ cap, we grew crystals of the catalytic domain of *S. pombe* aprataxin that had been preincubated with GMP, the product of the DNAppG de-capping reaction, and determined the structure at 1.5 Å resolution (Table 1). The crystals contained two aprataxin protomers in the asymmetric unit. Figure 3A shows a stereo view of the superimposed tertiary structures of the A and B protomers, which were virtually identical (0.51 Å rmsd at 192 Cα positions) except for a six amino acid disordered segment in a surface loop of the B protomer. As noted previously (9–11), aprataxin consists of two modules: a proximal HIT domain and a C-terminal zinc-finger. The zinc atom is colored green in Figure 3A and is coordinated with tetrahedral geometry by Cys200, Cys203, His217, and Glu221. The HIT domain is organized around a central 6-strand antiparallel β-sheet that, along with flanking loops and helices, forms the NMP-binding pocket. A simulated annealing F_o_–F_c_ omit electron density map verified the presence of a guanine nucleoside in the aprataxin active site (Supplementary Figure S1). The refined model included guanosine in the active site of the A protomer and GMP in the active site of the B protomer, in identical positions and *anti* nucleoside conformations (Figure 3A).

A detailed stereo view of the guanylate in the active site is shown in Figure 3B. The phosphate is flanked by the catalytic His138, His147, and His149 side chains. The planar purine ring of the guanine nucleobase is sandwiched by van der Waals contacts to Leu38 and Met64 (Figure 3B). The same purine non-specific hydrophobic contacts are seen in the aprataxin·AMP structures (10,11). The striking feature of the guanine-bound aprataxin is an extensive hydrogen bonding network to the O6, N1, N2 edge of the nucleobase. The Tyr41 hydroxyl engages in direct bifurcated hydrogen bonds to the guanine N1 and N2 atoms and in a water-bridged contact to the guanine O6 (Figure 3B). The guanine N2 atom is contacted by the Arg62 main-chain carbonyl and the Asn50 side chain (which in turn, makes bridging hydrogen bonds to Tyr41-OH and Arg62-O). By contrast, in the aprataxin·AMP complex, Tyr41 makes a single hydrogen bond to the adenine N1 atom and this is the only polar contact made by aprataxin to the adenine base edge (10,11). Indeed, in the AMP complex, the Asn50 side chain adopts a different rotamer that is oriented away from adenine base (10,11). It is apparent from the structure that the nucleotide-binding site would easily accommodate inosine, presumably maintaining the Tyr41 contacts to inosine O6 and N1. To our inspection, a 6-O-methyl group would not engender a steric clash.

Aprataxin makes multiple contacts to the vicinal hydroxyls of the cap ribose sugar. Asp63 is a bidentate acceptor of hydrogen bonds from the 2′-OH and 3′-OH groups. Lys67 makes a direct hydrogen bond to the 2′-OH and a water-mediated contact to the 3′-OH. The enzymic interactions with the ribose 2′-OH might explain why aprataxin displays feeble activity in removing a deoxyguanosine DNA 3′ cap.

### Effect of active site mutations on DNA 3′ de-capping activity

**Figure 4.**
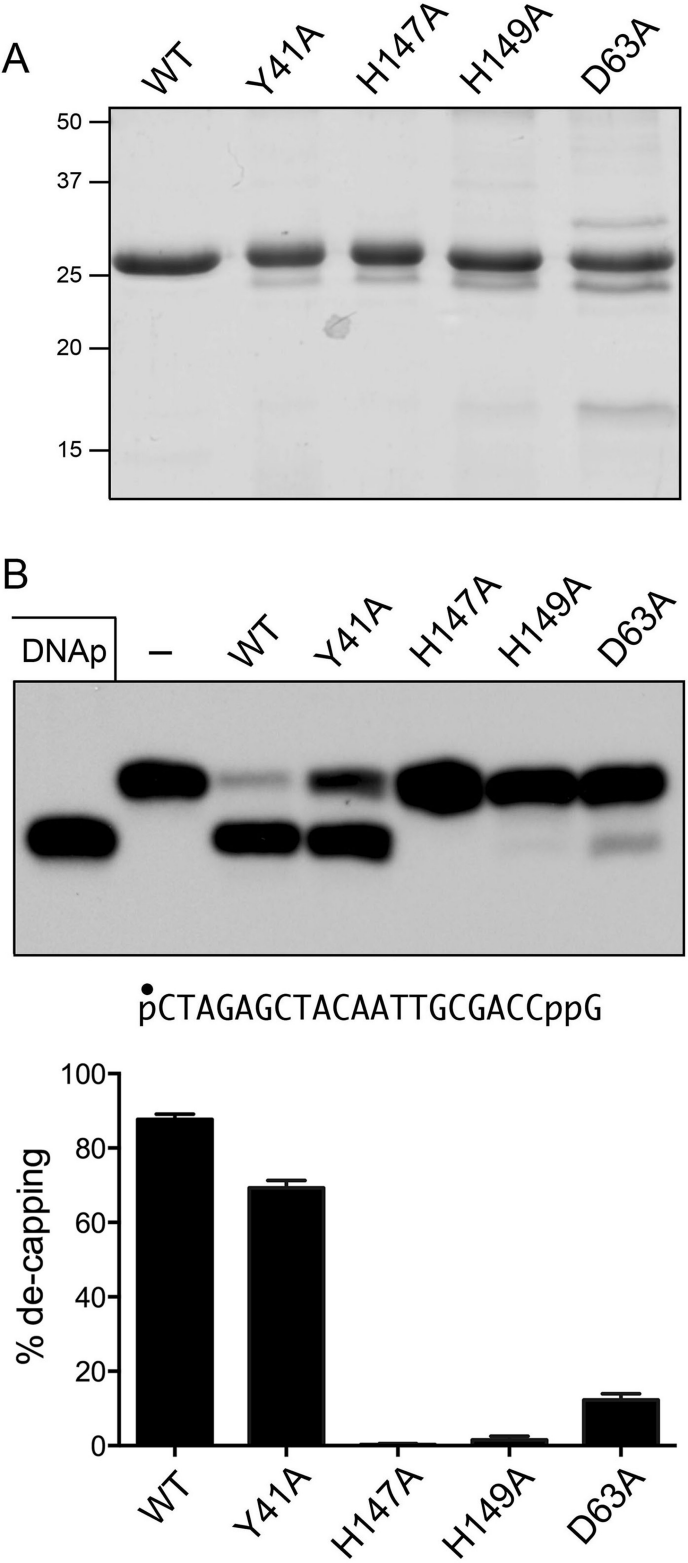
Effect of alanine mutations on de-capping. (**A**) Aliquots (5 μg) of purified recombinant wild-type (WT) aprataxin and the Y41A, H147A, H149A or D63A mutants were analyzed by SDS-PAGE. The Coomassie blue-stained gel is shown. The positions and sizes (kDa) of marker polypeptides are indicated at *left*. (**B**) Reaction mixtures (10 μl) containing 50 mM Tris–HCl (pH 8.0), 40 mM NaCl, 5 mM EDTA, 1 pmol 5′ ^32^P-labeled pDNAppG, and 16 pmol WT or mutant aprataxin as specified were incubated at 30°C for 10 min. The products were analyzed by urea-PAGE and visualized by autoradiography (top panel). The extents of de-capping are plotted in bar graph format (bottom panel). Each datum in the graph is the average of three independent experiments ± SEM.

Nucleotidyl transfer by aprataxin occurs via nucleophilic attack by His147 on the NMP phosphorus to form a covalent aprataxin-(His147)–NMP intermediate and expel a monophosphate-terminated polynucleotide. The nearby His149 side chain coordinates one of the non-bridging trigonal oxygens of the phosphorane transition state (9). The AppDNA de-adenylylation activity of aprataxin is abolished by a H147N mutation (10). We reasoned that if DNAppG de-capping adheres to the same mechanism, then de-capping ought to depend on His147 and His149. To address this point, we produced recombinant full-length H147A and H149A mutants in *E. coli* and purified them by Ni-agarose chromatography in parallel with full-length wild-type aprataxin (Figure 4A). When assayed for de-capping of a 5′ ^32^P-labeled pDNAppG substrate by a molar excess of input aprataxin that sufficed for 88% conversion of pDNAppG to pDNAp product (Figure 4B), we observed that the H147A mutant was catalytically inert and the H149A mutant effected de-capping of only 1% of the input substrate. We conclude that the 5′ de-adenylylation and 3′ de-guanylylation reactions of aprataxin follow the same catalytic mechanism.

To probe the contributions of cap ribose interactions, we produced and purified mutant D63A (Figure 4A) and found that loss of the Asp63 carboxylate was extremely deleterious, such that excess D63A de-capped only 12% of the input DNAppG substrate (Figure 4B). Thus, we see concordant effects of subtracting the ribose 2′-OH and a ribose-binding component of the active site.

Finally, we tested the effects of alanine substitution for Tyr41, a residue that interacts with the guanine nucleobase. The Y41A mutant retained substantial activity, de-capping 69% of the input substrate (Figure 4B). We surmise that the relatively mild effects of Y41A are buffered by the multiple residual atomic contacts to the guanine N2 amine.

### Efficiency of 5′ de-adenylylation versus 3′ de-guanylylation

**Figure 5.**
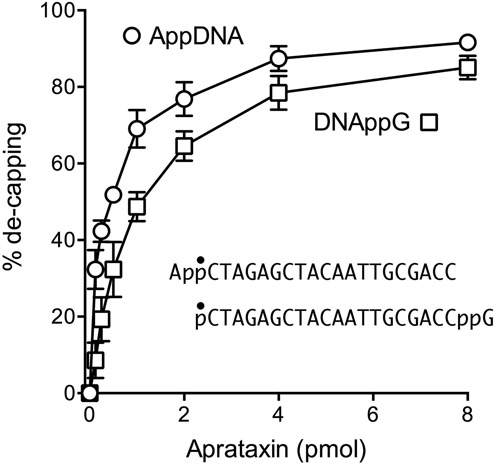
5′ de-adenylylation versus 3′ de-guanylylation. Reaction mixtures (10 μl) containing 50 mM Tris–HCl (pH 8.0), 40 mM NaCl, 5 mM EDTA, 0.2 pmol 5′ ^32^P-labeled AppDNA or pDNAppG substrates (of identical nucleobase sequence, as shown, with the radiolabeled phosphate denoted by •) and either 0.125, 0.25, 0.5 1, 2, 4, 8 pmol of aprataxin were incubated at 30°C for 10 min. The products were analyzed by urea-PAGE. The extents of conversion of pDNAppG to pDNAp and of AppDNA to pDNA are plotted as a function of input aprataxin. Each datum in the graph is the average of four or five independent experiments ± SEM.

In the experiment shown in Figure 5, we compared by enzyme titration the activity of aprataxin in de-capping a 20-mer AppDNA substrate versus a 20-mer DNAppG strand of identical nucleobase sequence. Whereas the extents of 5′ de-adenylylation and 3′ de-guanylylation were similar at saturating enzyme, the specific activity of aprataxin was 4-fold greater on the AppDNA substrate.

### Can aprataxin remove the guanylate cap from a DNAppG primer-template?

**Figure 6.**
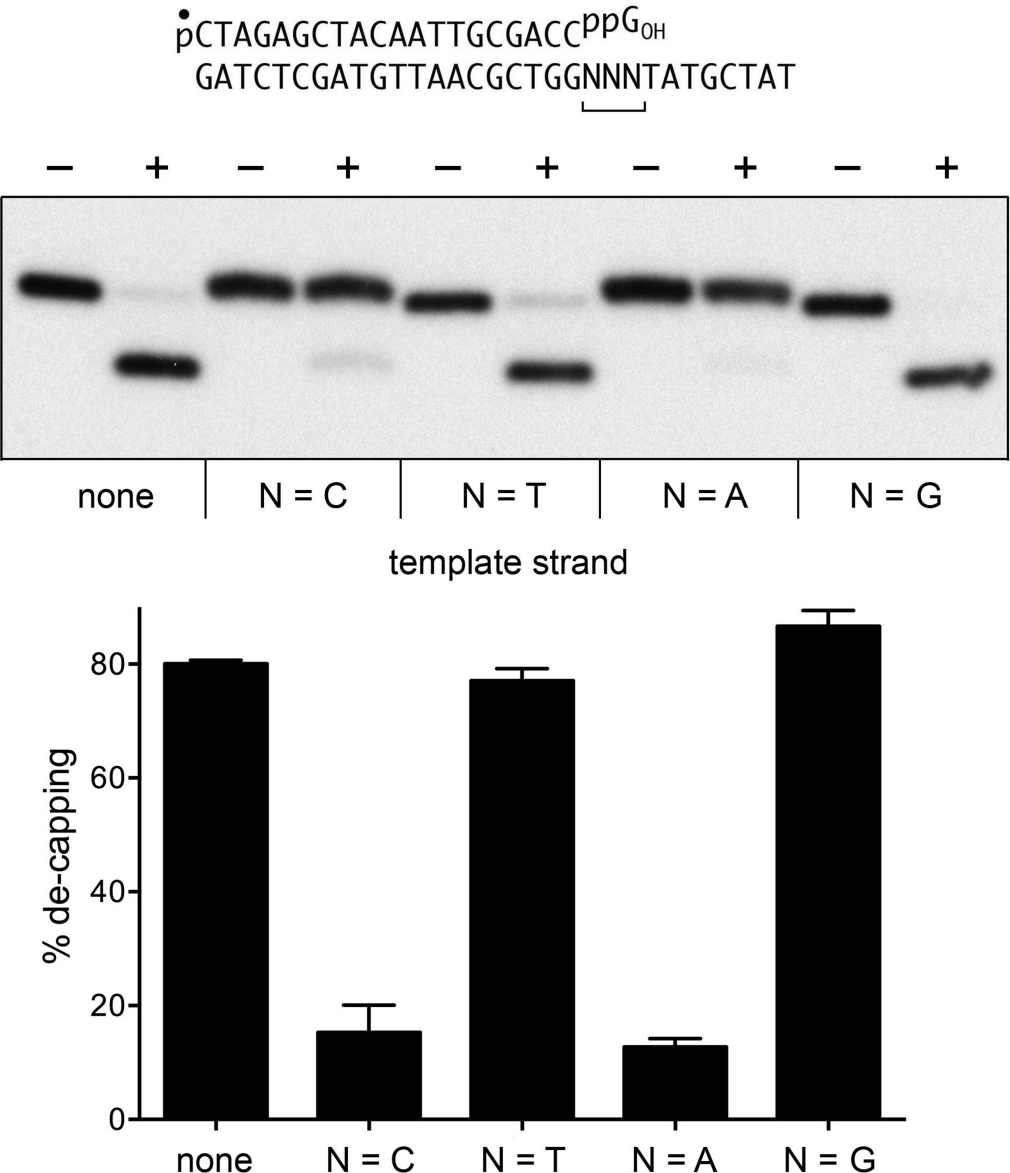
De-capping of DNAppG primer-templates. Reaction mixtures (10 μl) containing 50 mM Tris–HCl, pH 8.0, 40 mM NaCl, 5 mM EDTA, 0.2 pmol 5′ ^32^P-labeled pDNAppG (template ‘none’) or 5′ ^32^P-labeled pDNAppG primer-templates with CCC, TTT, AAA or GGG template trinucleotides flanking the primer terminus, and 16 pmol aprataxin (where indicated by +) were incubated at 30°C for 10 min. The products were analyzed by urea PAGE and visualized by autoradiography (top panel). The extents of conversation of pDNAppG to pDNAp for each substrate (±SEM) are plotted in bar graph format (bottom panel).

Guanylate capping of a recessed DNA 3′-PO_4_ end in duplex DNA creates an effective primer for repair synthesis by DNA polymerases, provided that the cap guanine nucleobase is positioned opposite a cytosine in the template strand. We reported that *E. coli* DNA polymerases I, II, III and human DNA polymerase β catalyzed no detectable nucleotide additions to a DNAppG_OH_ primer when the cap guanine is opposite T, A or G, i.e. they require a canonical G:C base pair for productive cap-primed synthesis (5). In principle, only one-fourth of the 3′ caps that might be formed at 3′-PO_4_ gaps in duplex DNA would serve as primers for DNA polymerases that display such fidelity. This scenario prompts two questions: (i) can aprataxin de-guanylylate a DNAppG primer-template substrate; and (ii) is de-capping influenced by the opposing template nucleobase. To address these issues, we prepared a series of 5′ ^32^P-labeled pDNAppG primer-templates that place the cap guanine across from a C, T, A, or G template base (Figure 6). The extent of aprataxin de-capping of a recessed DNAppG primer end opposite a template T or G nucleobase was similar to the extent of de-capping of a single-stranded DNAppG substrate (Figure 6). Aprataxin was much less adept at de-capping the primer strand when the cap guanine was opposite a C template nucleobase, suggesting that G:C pairing to the template shields the cap from aprataxin. (This is consistent with the available aprataxin·DNA·AMP structures, in which the AMP nucleotide is reflected away from the DNA duplex.) We were surprised to find that aprataxin de-guanylylation was similarly feeble when the cap guanine was opposite an A template nucleobase (Figure 6). We speculate that the pyrophosphate-linked guanosine might form a G:A mispair with the template strand.

### Attempt to crystallize aprataxin with 3′-PO_4_ DNA yields a complex in the 5′-OH orientation

We undertook crystallization trials of aprataxin that had been incubated with two complementary 10-mer DNA oligonucleotides, 5′ _HO_GAATCATAAC_OH_ and 5′ _HO_GTTATGATTCp, that were designed to form a 10-bp blunt duplex with a single 3′-terminal phosphate group. The goal was to capture a DNA complex with the 3′-PO_4_ strand poised at the active site, in a reverse orientation of the duplex *vis à vis* previous aprataxin·DNA complexes wherein the DNA 5′-OH terminus was at the active site (10,11). Crystals were obtained in space group P321. After molecular replacement with the aprataxin polypeptide, we were able to place a DNA oligonucleotide in electron density. The structure of the complex was refined at 2.25 Å resolution to R/R_free_ of 18.0/24.0 (Supplementary Table S1). The asymmetric unit contained one aprataxin protomer and one copy of the _HO_DNA_OH_ strand. A second copy of each was situated at a crystallographic two-fold, so that the two DNA strands formed a 10-nt blunt duplex, albeit with 8/10 base mismatches, the terminal G:C pair being the only one with canonical Watson–Crick configuration. An aprataxin protomer was engaged at each end of the duplex, with the 5′-OH terminus of the ‘substrate strand’ projecting toward the active site (Supplementary Figure S2A). The present aprataxin structure in complex with a 10-mer mispaired DNA duplex recapitulates the features of aprataxin in complex with a 13-mer perfectly paired DNA duplex (11), whereby one aprataxin protomer is bound at each end of the DNA; notwithstanding that the present and prior crystals were in different space groups and the second aprataxin protomers at the DNA ends were in quite different positions when of the first aprataxin protomers were superimposed (Supplementary Figure S2B).

**Figure 7.**
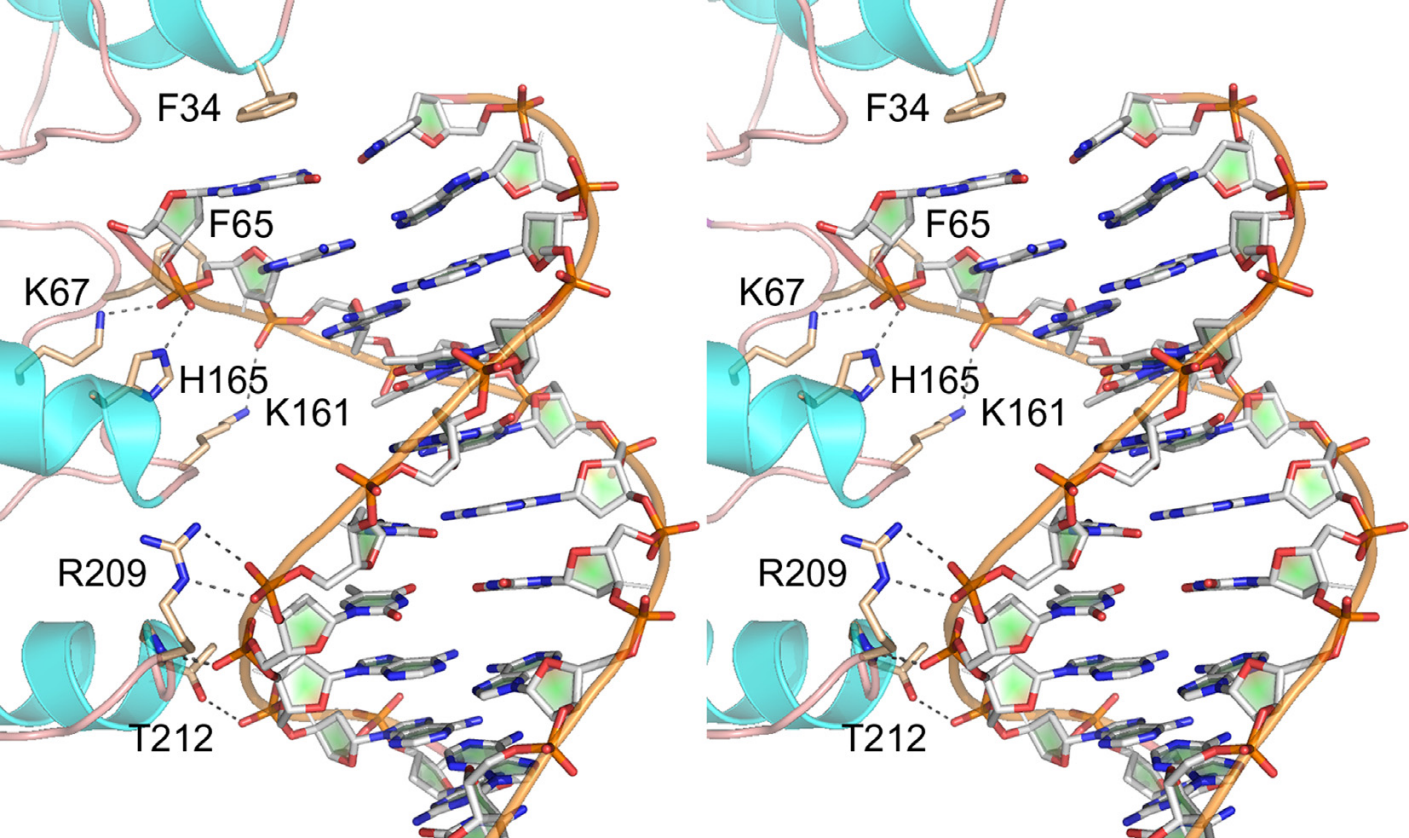
Aprataxin·DNA complex. Stereo view of the interface of aprataxin with the 10-mer DNA (stick model with gray carbons) in the presently solved complex in space group P321. Relevant protein side chain and main chain atoms are depicted as stick models with beige carbons. Atomic contacts to the phosphodiester backbone are denoted by dashed lines.

In brief, the aprataxin-DNA interface in the present structure spans 8-nt (Figure 7). The duplex end is bookmarked by Phe34, which makes a π-stack on the guanine nucleobase of the 5′ G:C base pair. Phe65 packs again the penultimate ribose of the substrate strand. All other contacts are with the phosphate backbone. Lys67 and His165 coordinate the terminal phosphodiester of the substrate strand and Lys161 coordinates the second phosphodiester. Distal atomic contacts to the template strand are via the Zn-finger domain. Arg209 makes a bidentate interaction with the sixth phosphodiester. The seventh phosphodiester is engaged by the Phe211 and Thr212 main-chain amides. The eight phosphodiester receives a hydrogen bond from by the Thr212 side chain (Figure 7).

### Concluding remarks

An early hypothesis that aprataxin's nucleotidyltransferase activity might be dedicated to hydrolysis of protein-(lysyl)-NMP adducts (17) was supplanted by compelling biochemical (8) and structural (9–11) evidence that AppDNA intermediates of abortive ligation are physiological substrates for aprataxin. Because classic DNA ligases exclusively transfer AMP to the 5′-PO_4_ DNA strand, there had been little attention paid to aprataxin's nucleobase specificity. We recently extended aprataxin's repertoire to the de-capping of DNAppG ends formed by the RNA ligase RtcB (4). Here, by taking advantage of RtcB's ability to utilize certain GTP analogs to synthesize DNAppN caps, we show that aprataxin hydrolyzes guanosine, inosine and 6-O-methylguanosine caps. The 1.5 Å structure of aprataxin with GMP in the active site rationalizes the range of acceptable purine nucleobases, while highlighting that the enzyme actually makes more extensive contacts with guanine than it does with adenine.

Aprataxin is not adept at de-capping deoxyguanosine, reflecting the importance of the ribose 2′-OH hydroxyl contacts seen in the crystal structure. We presume that aprataxin would have similarly feeble activity with a 5′ deoxyadenylylated (dA)ppDNA substrate. Whereas many ATP-dependent DNA ligases do not utilize dATP (18–20), there are some DNA ligases than can (21,22) and one presumes they could generate abortive (dA)ppDNA intermediates that would not be rectified by aprataxin. However, the risk of DNA ligases or RtcB generating deoxypurine blocked ends *in vivo* might be low, insofar as intracellular rNTP concentrations are much higher than dNTP concentrations (23).

The unique chemical mechanism of end joining by RtcB was suggested to be the bellwether of an alternative enzymology based on covalently activated 3′-PO_4_ ends as intermediates in the synthesis of polynucleotide 3′-5′ phosphodiesters (2,3). Covalent nucleotidylation of an RNA 3′-PO_4_ end to form RNA_3′_pp_5′_A was first described as an evanescent intermediate in the synthesis of an RNA 2′,3′-cyclic phosphate terminus by the enzyme RNA cyclase (RtcA), which transfers AMP from ATP via a covalent RtcA-(histidinyl)–AMP intermediate (24,25). Yet the RtcA repertoire also embraces highly efficient adenylylation of RNA and DNA 5′-PO_4_ ends to form stable AppRNA and AppDNA end products (26). Thus, there is considerable plasticity in the direction of the RtcA pathway (2′,3′ cyclization versus 5′ A capping), with attendant uncertainty as to what the ‘real’ substrates of RtcA are. This theme is amplified by the recent report of Zhelkovsky and McReynolds (27) that certain classic ATP-dependent thermophilic RNA ligases, which join 3′-OH/5′-PO_4_ ends via an AppRNA intermediate, are also capable of adenylylating a DNA 3′-PO_4_ end to form a stable DNAppA product. In effect, the thermophilic RNA ligases double as DNA 3′ capping enzymes, albeit with an A cap rather than a G cap. Following on our discovery of aprataxin as a DNAppG 3′ de-capping enzyme, they found that *Saccharomyces cerevisiae* aprataxin removes the A cap from DNAppA (27).

Although the prevalence of DNAppG and DNAppA caps *in vivo* is uncharted territory, it is conceivable that they contribute to the pathophysiology of aprataxin deficiency. It will be of acute interest to structurally illuminate the interface of aprataxin with a DNAppN substrate and to attempt to engineer separation-of-function mutations that selectively impact its activity at 5′ or 3′ capped ends. Our efforts here to capture a structure of aprataxin with a DNA 3′-PO_4_ in the active site were unfruitful. The structure we did obtain, in a 5′-OH orientation, echoes those reported previously (10,11) and underscores the theme that fission yeast aprataxin favors crystallization with the enzyme bound to both 5′-OH duplex ends, seemingly independent of a specific duplex length or a requirement for canonical nucleobase pairing.

## SUPPLEMENTARY DATA

Supplementary Data are available at NAR Online.

SUPPLEMENTARY DATA
