# Supplementary material for: DNA3′pp5′G de-capping activity of aprataxin: effect of cap nucleoside analogs and structural basis for guanosine recognition
Source: Nucleic Acids Res. 2015 May 24;43(12):6075–83. doi: 10.1093/nar/gkv501 (PMC4499129; doi:10.1093/nar/gkv501)
Supplement: SUPPLEMENTARY DATA [file supp_43_12_6075__index.html]

DNA3′pp5′G de-capping activity of aprataxin: effect of cap nucleoside analogs and structural basis for guanosine recognition — DNA3′pp5′G de-capping activity of aprataxin: effect of cap nucleoside analogs and structural basis for guanosine recognition — SUPPLEMENTARY DATA 

# DNA3′pp5′G de-capping activity of aprataxin: effect of cap nucleoside analogs and structural basis for guanosine recognition

## SUPPLEMENTARY DATA

- SUPPLEMENTARY DATA
