# Supplementary material for: DNA3′pp5′G de-capping activity of aprataxin: effect of cap nucleoside analogs and structural basis for guanosine recognition
Source: Nucleic Acids Res. 2015 May 24;43(12):6075–83. doi: 10.1093/nar/gkv501 (PMC4499129; doi:10.1093/nar/gkv501)
Supplement: SUPPLEMENTARY DATA [file supp_gkv501_nar-00679-h-2015-File009.pdf]

Supplemental Material

$DNA_{3'}pp_{5'}G$ de-capping activity of aprataxin: effect of cap nucleoside analogs and structural basis for guanosine recognition

Mathieu Chauleau, Agata Jacewicz, and Stewart Shuman

SUPPLEMENTAL METHODS

Structure of an aprataxin•DNA complex. 2 µl of a solution containing 0.6 mM fission yeast aprataxin-(33-232), 5 mM guanosine, and 1.2 mM each of complementary 10-mer DNA oligonucleotides 5' $_{HO}$GTTATGATTCp and 5' $_{HO}$GAATCATAAC$_{OH}$ was mixed with 2 µl of precipitant solution containing 15% (w/v) PEG-3350, 0.15 M magnesium acetate. Crystals were grown at 22˚C by sitting drop vapor diffusion against a reservoir of the precipitant solution. Crystals appearing after 3 days were cryoprotected by transfer to a solution containing 25% glycerol, 25% (w/v) PEG-3350, 0.15 M magnesium acetate prior to flash-freezing in liquid nitrogen. Diffraction data at 2.25 Å resolution were collected from a single crystal at the Argonne National Laboratory beamline ID-24-C equipped with Pilatus 6M-F detector. The crystal belonged to space group P321 with unit cell dimensions consistent with one protomer per asymmetric unit, assuming a solvent content of 62%. Indexing, merging and scaling of the diffraction data were performed in XDS and Aimless. The structure was solved by molecular replacement in Phenix.Phaser using as a search model the *S. pombe* Aprataxin-(33-332) polypeptide structure reported presently, from which GMP and waters were removed. The asymmetric unit contained one aprataxin protomer. The structure was iteratively refined in Phenix and adjusted manually in Coot. The aprataxin protomer comprised two segments, from 33 to 187 and 191 to 230. Difference density allowed placement of one 10-mer $_{HO}$GAATCATAAC$_{OH}$ DNA strand in the asymmetric unit. Guanosine was placed into density in the active site. A second aprataxin•oligonucleotide complex was situated at a crystallographic two-fold symmetry axis, so that the two strands formed a 10-nucleotide DNA duplex with 8/10 base mismatches, the terminal G:C pair being the only one with canonical Watson-Crick configuration. An aprataxin protomer is engaged at each end of the duplex, with the 5'-OH terminus of the "substrate strand" projecting toward the active site. The final 2.3 Å model was refined to R/R_{free} of 18.0/24.0 with no Ramachandran outliers (Table S1). The coordinates have been deposited in the Protein Data Bank under accession code 4YKL.

Table 1: **Diffraction data and refinement statistics**

**Diffraction data**	
Space group	*P321*
Cell dimensions	
a, *b*, *c* (Å)	119.1, 119.1, 35.6
α, β, γ (˚)	90.0, 90.0, 120.0
Wavelength (Å)	1.77
Resolution (Å)	59.54−2.25 (2.32−2.25)
Reflections	14369
observed	161422 (13822)
unique	14369 (1310)
R_{merge} [a]	0.077 (0.747)
R_{meas} [b]	0.080 (0.786)
R_{pim} [c]	0.024 (0.241)
I / σI	20.7 (3.6)
CC(1/2) [d]	0.999 (0.916)
Completeness (%)	100.0 (100.0)
Multiplicity	11.2 (10.6)
Wilson B-factor	40.0
**Refinement statistics**	
Resolution (Å)	59.56−2.25 (2.39−2.25)
Completeness	100.0 (99.9)
R_{work} / R_{free} [e]	18.0 / 24.0
CC* [d]	1.000 (0.976)
RMS deviations	
Bond lengths (Å)	0.008
Bond angles (˚)	1.188
Ramachandran plot	
Favored (%)	95.8
Outliers	none
**Model contents**	
Protomers / ASU	1
Protein residues	195
Water	53
Ligands/Ions	DNA oligonucleotide 5'-GAATCATAAC, 1 guanosine, 4 glycerol, 2 Cl^-, 1 Zn^{2+}
**PDB ID**	4YKL

Figures in parentheses refer to data in the highest resolution shell. Data collection statistics are from Aimless (CCP4 suite), refinement and geometric statistics come from Phenix.Refine.

a) R_{merge} describes the spread of multiple observations of the intensity of the unique reflections.

b) R_{meas} (the redundancy-corrected R_{merge}) indicates the precision of an individual intensity measurement independently of the multiplicity of that measurement.

c) R_{pim} reports the precision of an averaged intensity measurement.

d) CC(1/2) and CC* are statistics calculated for defining the high-resolution cutoff limits [CC(1/2)] and the quality of the diffraction data in the context of the refined structure [CC*].

e) R_{free} set consists of 6% of data chosen randomly against which structure was not refined.

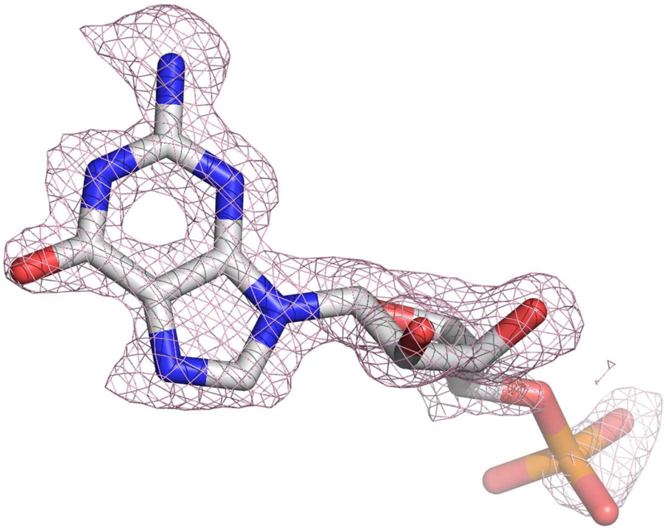

Figure S1. **Electron density for the cap guanosine**. A simulated annealing Fo-Fc omit electron density map (mesh) of the nucleoside in the active site of aprataxin, contoured at 5 σ.

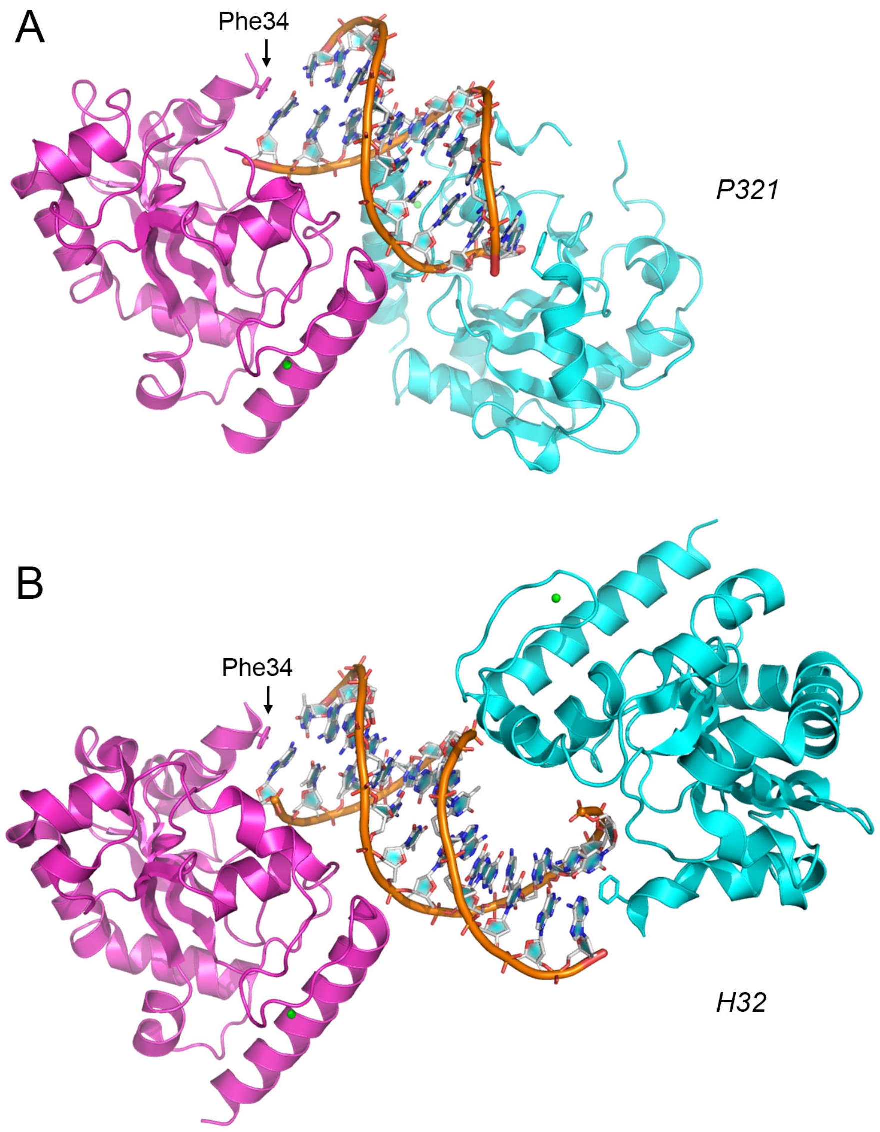

Figure S2. **Aprataxin•DNA complexes**. The presently solved structure of fission yeast aprataxin bound to a 10-mer DNA oligonucleotide in space group P321 (panel A) was aligned to the structure of fission yeast aprataxin bound to a 13-bp duplex in space group H32 (pdb ID: 3SPL) (panel B). In each case, an aprataxin protomer is engaged at the 5' end of the substrate strand such that Phe34 stacks on the terminal nucleobase. The structures were superimposed with respect to the magenta protomers. The zinc atoms are depicted as green spheres.
